# Content Quality of YouTube Videos About Pain Management After Cesarean Birth: Content Analysis

**DOI:** 10.2196/40802

**Published:** 2023-06-23

**Authors:** Natalie A Squires, Elizabeth Soyemi, Lynn M Yee, Eleanor M Birch, Nevert Badreldin

**Affiliations:** 1 Department of Obstetrics and Gynecology New York Presbyterian Hospital Weill Cornell Medical Center New York, NY United States; 2 Illinois Math and Science Academy Aurora, IL United States; 3 Division of Maternal-Fetal Medicine Department of Obstetrics and Gynecology Northwestern University Feinberg School of Medicine Chicago, IL United States; 4 Department of Emergency Medicine Madigan Army Medical Center Joint Base Lewis-McChord, WA United States

**Keywords:** health information, internet, YouTube, cesarean section, cesarean, C-section, postpartum, social media, web-based video, maternal, postnatal, pain, systematic search, patient education, information quality, accuracy, credibility, health education, educational video, education resource, health video

## Abstract

**Background:**

YouTube is an increasingly common source of health information; however, the reliability and quality of the information are inadequately understood. Several studies have evaluated YouTube as a resource during pregnancy and found the available information to be of poor quality. Given the increasing attention to postpartum health and the importance of promoting safe opioid use after birth, YouTube may be a source of information for birthing individuals. However, little is known about the available information on YouTube regarding postpartum pain.

**Objective:**

The purpose of this study is to systematically evaluate the quality of YouTube videos as an educational resource for postpartum cesarean pain management.

**Methods:**

A systematic search of YouTube videos was conducted on June 25, 2021, using 36 postpartum cesarean pain management–related keywords, which were identified by clinical experts. The search replicated a default YouTube search via a public account. The first 60 results from each keyword search were reviewed, and unique videos were analyzed. An overall content score was developed based on prior literature and expert opinion to evaluate the video’s relevance and comprehensiveness. The DISCERN instrument, a validated metric to assess consumer health information, was used to evaluate the reliability of video information. Videos with an overall content score of ≥5 and a DISCERN score of ≥39 were classified as high-quality health education resources. Descriptive analysis and intergroup comparisons by video source and quality were conducted.

**Results:**

Of 73 unique videos, video sources included medical videos (n=36, 49%), followed by personal video blogs (vlogs; n=32, 44%), advertisements (n=3, 4%), and media (n=2, 3%). The average overall content score was 3.6 (SD 2.0) out of 9, and the average DISCERN score was 39.2 (SD 8.1) out of 75, indicating low comprehensiveness and fair information reliability, respectively. High-quality videos (n=22, 30%) most frequently addressed overall content regarding pain duration (22/22, 100%), pain types (20/22, 91%), return-to-activity instructions (19/22, 86%), and nonpharmacologic methods for pain control (19/22, 86%). There were differences in the overall content score (*P=*.02) by video source but not DISCERN score (*P=*.45). Personal vlogs had the highest overall content score at 4.0 (SD 2.1), followed by medical videos at 3.3 (SD 2.0). Longer video duration and a greater number of comments and likes were significantly correlated with the overall content score, whereas the number of video comments was inversely correlated with the DISCERN score.

**Conclusions:**

Individuals seeking information from YouTube regarding postpartum cesarean pain management are likely to encounter videos that lack adequate comprehensiveness and reliability. Clinicians should counsel patients to exercise caution when using YouTube as a health information resource.

## Introduction

YouTube is a frequently visited website in the United States and a common source of eHealth information [1,2]. As an alternative to written communication, YouTube provides an opportunity to narrow the health literacy gap if quality health information is presented clearly and comprehensively [3]. Indeed, studies have demonstrated that some patients prefer video over written sources of medical information [4]. However, accessing YouTube for health information remains problematic, as there are few regulations governing the information available.

Recent studies have evaluated YouTube as a source of health information during pregnancy. Chandrasekaran et al [5] evaluated the use of various social media platforms as a resource for the Zika virus. The authors found that while YouTube provided a similar number of informative results when compared to other platforms, it also included a higher number of outdated and misleading results, including hoax messages and conspiracy theories [5]. Similarly, YouTube videos discussing medication use in pregnancy were found to have inconsistent or inadequate safety information [6].

Pain is a significant concern among postpartum individuals [7,8]. Inadequately controlled pain in the early postpartum period increases individuals’ risk of experiencing persistent pain, depressive symptoms, and opioid abuse [9,10]. As such, practice guidelines make a strong recommendation for patient education and antenatal counseling regarding postpartum pain management protocols to optimize their recovery [11]. However, the optimal mode and content of this counseling have not been established.

Given the unique challenges of the early postpartum period, individuals may use internet resources to address concerns related to their postcesarean birth pain and recovery. While the growing popularity of YouTube has the possibility to improve access to postpartum care and the postpartum pain experience, there are limited data evaluating the quality of available resources for postpartum pain management. Thus, the purpose of this study was to systematically evaluate the quality of YouTube videos on postpartum cesarean recovery.

## Methods

### Search Strategy

A systematic search of YouTube videos was conducted using postpartum cesarean pain management–related keywords on June 25, 2021. Search terms were identified by expert consensus and expanded using Google Trends to identify related searches. The final terms included 36 iterations of the search “postpartum cesarean pain” (Multimedia Appendix 1).

To duplicate a public search, the search was performed in incognito mode in a cache-cleared browser, and no registered account was used. Search results were sorted by relevance, which is the default setting for YouTube searches. The first 60 results from each keyword search were collected, and duplicates were omitted. This sort of strategy and sample size were selected based on data showing that 83% of searchers will not view more than three web pages of results [12]. Videos were excluded if the full video was unavailable, was >30 minutes in duration, or was in a language other than English. Video duration was capped based on research showing that web search queries for adults were on average 18 minutes in duration, and thus longer videos are unlikely to be viewed by the general public [13]. The remaining videos were assessed for inclusion by screening the video titles, comments, and channels for terms related to postcesarean pain. If any uncertainty remained, videos less than 10 minutes in duration were watched in their entirety. If videos were longer than 10 minutes, the first 10 minutes were watched, and the reviewer reviewed additional time stamps or sections indicating a shift in content to verify eligibility. This process was designed to mirror that of a traditional systematic review, wherein a sample of the content (ie, abstracts) is initially reviewed to determine relevance prior to the review of the full content. Videos were also excluded if the content was unrelated to cesarean delivery, postpartum pain management, or recovery (ie, if the overall content score was 0, as described below).

### Data Extraction

Descriptive characteristics of each video were gathered, including the date posted, video length, number of comments, likes, dislikes, and channel subscribers. Values that accumulate over time were collected within one day (July 12, 2021) by a single reviewer to minimize variability. Video source and presenter characteristics were also collected. Video sources were categorized as personal video blogs (vlogs), medical or hospital videos, advertisements, and media. The source was determined based on the affiliation of the video author and the channel description, when applicable. Videos were labeled as vlogs when the video author had an independent channel describing their personal experience and recommendations. Medical or hospital videos were differentiated by a clear affiliation with a hospital or medical service company. Video bloggers who identified as medical professionals on their independent channels were characterized as personal vloggers. Advertisement videos differed from medical videos in that they clearly described the benefits of a single product in the postpartum period. Media videos included news clips and talk show interviews. Videos were labeled as character videos if a specific, identifiable person presented the information. Presenter characteristics were identified when applicable, and reviewers subjectively identified the presenter’s gender, race or ethnicity, and age.

### Content Analysis

Two content scores were developed using the expert opinions of maternal-fetal medicine specialists (NB and LMY) in conjunction with American College of Obstetrician and Gynecologists guidelines regarding pain management [14]. Both scores were used to evaluate the video’s relevance and comprehensiveness as a health education resource. The first, an “overall content score,” included nine topics relevant to postcesarean pain management: (1) pain duration, (2) pain types, (3) when to notify a clinician, (4) activity recommendations, (5) pain medication timing, (6) multimodal pharmacologic methods, (7) nonpharmacologic methods, (8) maternal risks of treatments, and (9) risks to newborns. Second, given growing awareness regarding opioid use in the postpartum period, a second “opioid content score” was used to evaluate the comprehensiveness with regard to opioid use in postpartum pain management. This was scored based on the following nine topics: (1) addressing opioid use, (2) when to use, (3) limitations of use, (4) general maternal risks of treatment, (5) risk of addiction, (6) risks to newborns, (7) length of use, (8) discharge instructions, and (9) disposal of remaining tablets. For each of the content scores, one point was awarded if a topic was mentioned, for a total possible score of 9. Higher content scores indicated greater comprehensiveness in the video. Similar content assessments have been used in prior studies to evaluate YouTube as a health information resource [15,16].

### DISCERN Analysis

The DISCERN instrument was used to assess the quality and reliability of the videos as an information source. This tool has been widely used to evaluate web-based sources of health information, including YouTube videos [16-21]. Studies have demonstrated that the DISCERN tool enables both professionals and consumers to distinguish between high- and low-quality sources of health information [19,22]. The DISCERN instrument consists of 15 questions plus an overall quality rating to assess consumer health information on treatment choices. The first 8 questions address the reliability or trustworthiness of a source, followed by 7 questions evaluating whether consumers had access to detailed information regarding their treatment options. Questions are rated on a Likert scale of 1-5, with a score of 1 indicating the criterion was not satisfied and a score of 5 indicating the criterion was fully satisfied. Specific guidelines on the application scoring criteria are provided via the Online Discern Tool [23]. Like prior studies, we report the DISCERN score as a sum of the first 15 questions, and the score was interpreted with established categories describing source reliability: excellent (63-75 points), good (51-62), fair (39-50), poor (28-38), or very poor (≤27) [20,21,24].

### Quality Analysis

A combination of the overall content score and the DISCERN score was used to establish video quality as a health education resource that is both comprehensive and reliable. Videos with an overall content score of ≥5 and DISCERN score of ≥39 were classified as high-quality*.* These criteria were chosen as a DISCERN score of ≥39 indicates at least fair information reliability, and an overall content score of ≥5 indicates that greater than half of the content criteria were met.

Consensus regarding the application of scoring criteria was obtained through a collaborative review of 3 videos among 3 authors (NS, ES, and NB). Subsequently, the application of the scoring criteria was tested via an independent review of 10 videos. The average DISCERN scoring disparity was 0.18 points. Intraclass correlation was 0.76 and interclass correlation was 0.81, indicating good interrater reliability. Areas of discordance were resolved by team discussion. The remaining videos were divided and scored by authors NS or ES. Data were extracted and stored using REDCap (Research Electronic Data Capture; Vanderbilt University) software.

### Statistical Analysis

All statistical analysis was performed using Excel software (version 16.56, Microsoft Corp). Interrater agreement was analyzed by intraclass correlation coefficients and a single-factor ANOVA. Video characteristics were analyzed via descriptive statistics. Associations among video source, quality, and descriptive characteristics were evaluated using nonparametric correlations. A *P* value of less than .05 was considered significant.

### Ethics Approval

This study does not involve human subject research and was deemed exempt by Northwestern University’s institutional review board (reference number: STU00214706).

## Results

### Video Characteristics

**Figure 1:**
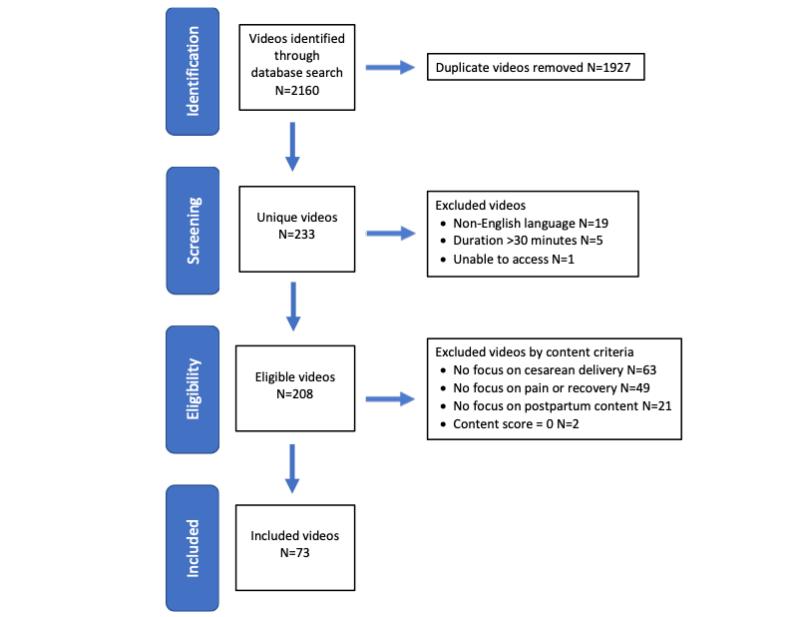
YouTube video selection regarding postcesarean pain management. The figure illustrates a flow diagram of the identification, selection, and exclusion of YouTube videos. The first 60 video titles for 36 unique search terms were collected for a total of 2160 videos. 73 videos were included for the final analysis.

A total of 233 unique videos were identified. Following the application of inclusion and exclusion criteria, 73 videos remained for analysis (Figure 1, Multimedia Appendix 2). Most videos (69/73, 95%) were character videos. Among these, most presenters appeared to be female (63/69, 91%), of reproductive age (56/69, 81%), and non-Hispanic White race or ethnicity (39/69, 57%). Video sources were most commonly medical videos (36/73, 49%), followed by personal vlogs (32/73, 44%), advertisements (3/73, 4%), and media (2/73, 3%; Table 1). The median length of videos was 8.03 minutes and they were uploaded for a median of 1230 days at the time of access (Table 1).

**Table 1:** Characteristics of videos included in review (N=73).

Characteristics		Values
Character video, n (%)		69 (95)
**Presenter age,** **n (%)**		
	Adolescent	1 (1)
	Reproductive age	58 (84)
	Older adult	10 (14)
**Presenter race or ethnicity, n (%)^a^**		
	Black	3 (4)
	White	44 (64)
	Latinx	1 (1)
	Asian	17 (25)
	Undetermined	4 (6)
**Video source,** **n (%)**		
	Medical	36 (49)
	Personal vlog	32 (44)
	Advertisement	3 (4)
	Media	2 (3)
**Video characteristics, median (IQR)**		
	Days since post	817 (551-1853)
	Duration (minutes)	7.8 (3.0-13.7)
	Views	40614 (6841-82748)
	Comments	15 (0-54)
	Likes	213 (53-902)
	Dislike	11 (3-34)
	Channel subscriber number	35100 (9020-177000)

^a^Presenter age and race or ethnicity were subjectively assigned.

### Content Analysis

**Figure 2:**
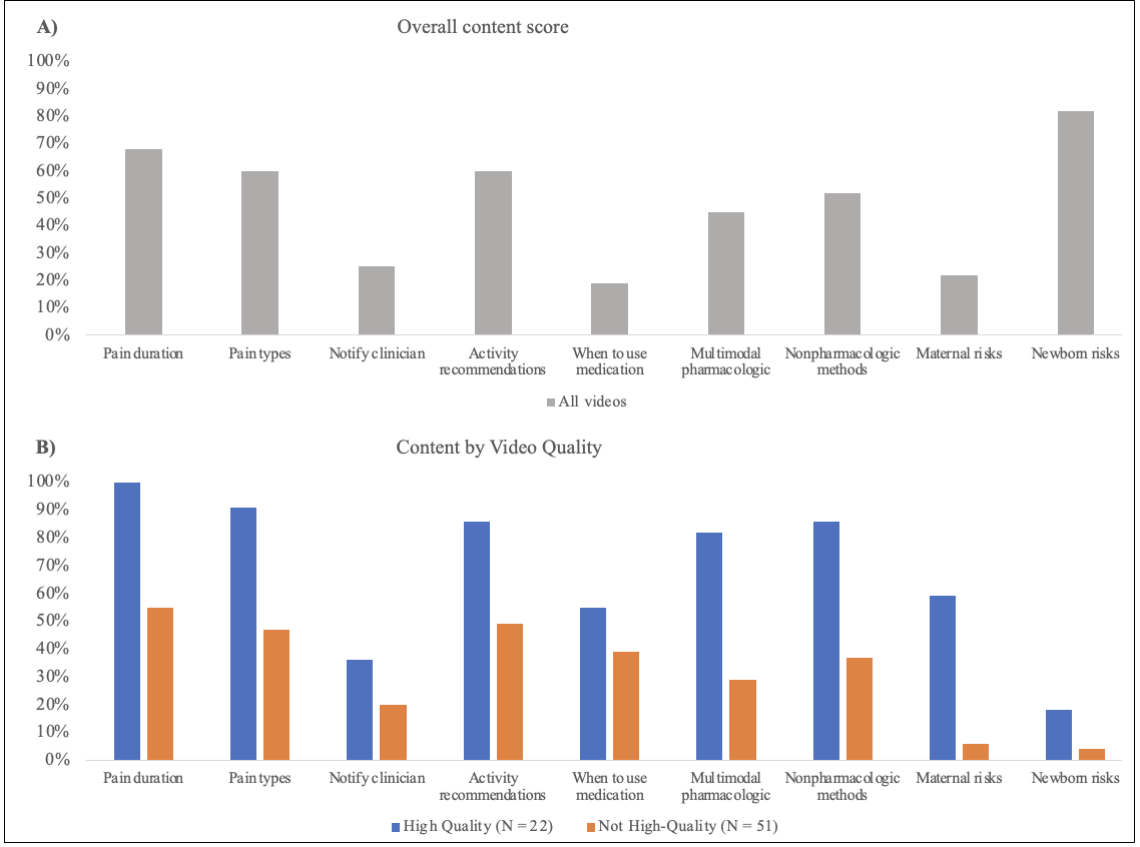
YouTube video content inclusion by topic area. The figure illustrates the overall content score by topic area. The y-axis demonstrates the percentage of total videos covering each of the 9 total topic areas. (A) The percentage of videos covering each topic area from all videos. (B) The percentage of videos by quality designation covering each topic area.

Regarding the overall content score, videos most frequently covered the expected duration of pain (50/73, 68%), different types of pain (44/73, 60%), and return to activity (44/73, 60%), whereas information on when to use medication (14/73, 19%) and risks to the newborn (6/73, 8%) were less frequently included (Figure 2). The mean overall content score was 3.6 (SD 2.0) out of 9. The overall content score significantly differed by the video source (*P=*.02). Personal vlog videos had the highest overall content score at 4.0 (SD 2.1), followed by medical videos at 3.3 (SD 2.0; Table 2).

Most videos (57/73, 78%) did not specifically address opioids and, therefore, had an opioid content score of 0. For those videos that did address opioids (16/73, 22%), videos most often covered maternal risks (9/16, 56%), limitations of opioids (7/16, 44%), and when to use opioids (6/16, 38%). Videos rarely discuss the risk of addiction (2/16, 13%), the recommended duration of use (1/16, 6%), or proper opioid disposal (0/16, 0%). For videos that addressed opioids, the mean opioid content score was 3.1 (SD 1.6) out of 9. There was no difference in opioid content score by video source (*P=*.77; Table 3).

**Table 2:** Quality of postpartum pain management videos on YouTube by video source.^a,b^

Video source	Overall content score			DISCERN score	
	Mean (SD)	*P* value	Mean (SD)		*P* value
Total (N=73)	3.6 (2.0)	N/A^c^	3.2 (8.1)		N/A
Medical (n=36)	3.3 (2.0)	.02	39.4 (8.7)		.45
Personal vlog (n=32)	4.0 (2.1)	N/A	38.9 (7.5)		N/A
Advertisement (n=3)	2.0 (0)	N/A	35.7 (8.6)		N/A
Media (n=2)	2.5 (0.7)	N/A	46 (5.6)		N/A

^a^The “overall content score” assesses video comprehensiveness related to postcesarean pain and is scored out of a maximum of 9 points.

^b^The DISCERN instrument evaluates the reliability of consumer health information. Higher scores indicate greater reliability. Scores are reported out of a maximum of 75. The following categories were used for score interpretation: excellent (63-75 points), good (51-62), fair (39-50), poor (28-38), and very poor (≤27).

^c^N/A: not applicable.

**Table 3:** Opioid content score by video source.^a,b^

Video source	Opioid content score	
	Mean (SD)	*P* value
Total (N=16)	3.1 (1.6)	N/A^c^
Medical (n=9)	2.9 (1.7)	.77
Personal vlog (n=5)	3.4 (1.8)	N/A
Advertisement (n=0)	—^d^	N/A
Media (n=2)	3.5 (0.7)	N/A

^a^The “opioid content score” assesses video comprehensiveness related to postcesarean opioid use and is scored out of a maximum of 9.

^b^Videos with an “opioid content score=0” were excluded from the analysis.

^c^N/A: not applicable.

^d^Not available.

### DISCERN Analysis

The DISCERN scores ranged from 22 (very poor reliability) to 59 (good reliability), with a mean DISCERN score of 39.2 (SD 8.1), consistent with fair reliability. No videos met the criteria for excellent reliability. The overall DISCERN score did not significantly differ by video source (*P=*.45; Table 2). Videos received the highest average score for DISCERN question 2, “Does it achieve its aims?” (mean 3.4), and question 3, “Is it relevant?” (mean 3.5). Videos received the lowest score for DISCERN question 4, “Does it provide sources?” (mean 1.8), and question 11, “Does it describe the risks of each treatment?” (mean 1.7; Multimedia Appendix 1).

Of the video characteristics, video duration (*r*=0.38; *P*<.01), the number of comments (*r*=0.30; *P*<.01), and the number of likes (*r*=0.32; *P*<.01) were significantly correlated with the overall content score. The number of comments was inversely correlated with the DISCERN score (*r*=–0.40; *P*<.01). No video characteristics were significantly correlated with the opioid content score (Table 4).

**Table 4:** Association of YouTube video comprehensiveness and reliability with video characteristics.

Video characteristics	Median (IQR)	Overall content score		Opioid content score			DISCERN score	
		Correlation (*r*)	*P* value	Correlation (*r*)	*P* value	Correlation (*r*)		*P* value
Days since post	817 (551-1853)	–0.01	.96	0.01	.95	–0.14		.25
Duration (minutes)	7.8 (3.0-13.7)	0.38	<.001	0.05	.64	<0.1		.94
Views	40,614 (6841-82,748)	0.20	.10	0.01	.94	–0.18		.14
Comments	15 (0-54)	0.30	.009	0.07	.82	–0.40		<.001
Likes	213 (53-902)	0.32	.005	0.03	.99	–0.08		.49
Dislike	11 (3-34)	0.17	.14	<–0.01	.56	–0.19		.10
Channel subscribers	35,100 (9020-177,000)	0.06	.59	–0.08	.49	0.13		.28

### Quality Analysis

A minority of videos (22/73, 30%) met the criteria for high quality. High-quality videos most frequently addressed overall content regarding pain duration (22/22, 100%) and pain types (20/22, 91%; Figure 2). High-quality videos infrequently address when to notify a clinician (8/22, 36%), and the risks of treatment to the newborn (4/22, 18%). Like trends for the overall content score, high-quality videos had significantly greater median video duration (13.0 minutes vs 7.6 minutes; *P=*.03), number of comments (24 vs 6; *P=*.04), and number of likes (397 vs 159; *P=*.04; Table 5). High-quality videos received the highest score for DISCERN question 2, “Does it achieve its aims?” (mean 3.9) and question 3, “Is it relevant?” (mean 4.1; Multimedia Appendix 3).

**Table 5:** YouTube video characteristics by quality designation.^a^

Video characteristics	High quality (N=22), median (IQR)	Not high quality (N=51), media (IQR)	*P* value
Days since post	738 (458-2220)	846 (551-1788)	.54
Duration (minutes)	13.0 (5.5-16.6)	7.6 (2.7-11.7)	.03
Views	53,865 (13,817-96,211)	29,661 (4857-75,572)	.20
Comments	24 (11-77)	6 (0-46)	.04
Likes	397 (210-992)	159 (32-632)	.04
Dislikes	20 (3-37)	10 (1-33)	.28
Subscribers	53,900 (9940-240,000)	32,900 (10,970-126,000)	.85

^a^High-quality videos were defined as videos with DISCERN scores greater than or equal to 39 and covering at least five topics out of 9 on the content score.

## Discussion

### Principal Results

In this study of the top 73 YouTube videos on postcesarean pain management, average video comprehensiveness was low and reliability was fair. Videos rarely address the full scope of health education topics relevant to preparing patients for their postcesarean pain experience. Interestingly, a greater number of comments and likes was positively correlated with better overall content, although more comments were also associated with poorer reliability according to the DISCERN instrument. These findings suggest greater video comprehensiveness is not necessarily associated with improved video reliability, and vice versa. Furthermore, only a minority of videos met the criteria for a high-quality health education resource, suggesting the information currently available on YouTube for postcesarean individuals has important limitations.

### Limitations

Like all web content, YouTube is a dynamic source of information. The search results in this study are limited in that they represent a cross-sectional sample. Additionally, the search strategy using the filter “relevance,” the default search setting on YouTube, represents only one filter method available to users. We used 36 different search terms to capture relevant videos; however, a different filter setting or search term may yield different findings. However, the chosen search terms were purposefully specific to established content criteria. The limited sample size may limit the ability to detect relationships between video characteristics and quality. Limitations exist in our screening process, where videos longer than 10 minutes were not watched in their entirety. It is possible that relevant content was missed using this strategy. Furthermore, our evaluation of the presenter’s characteristics (age, gender, and race or ethnicity) was limited by the fact that YouTube presenters rarely provide self-identifying information. Though our assessment was subjective, we felt that it was important to note representation, as this may influence viewership. Finally, our assessment of video quality was subjective. While we recognize the possibility that YouTube videos may be purposefully narrow in scope with high reliability, we purposefully defined high-quality videos as those that presented both comprehensive and reliable health information to the public.

### Comparison With Prior Work

Studies have evaluated eHealth information on pain management outside of the obstetrical population. In one study, the average DISCERN score for chronic pain websites was 55.9 out of a possible 80 points [20], suggesting that written resources may have a higher level of information reliability. However, there is a growing body of evidence that patient comprehension and satisfaction may improve with video over written resources [25,26]. These findings may be related to the average readability of written content. Despite recommendations that written patient education material be at a sixth grade reading level [27,28], the average readability of websites on chronic pain management was that of a 10th-11th grade student [20]. Another study found that web-based patient education materials across obstetric and gynecologic societies ranged from a 9th to 12th grade reading level [27,29]. Thus, video resources have the opportunity to minimize literacy as a barrier to obtaining reliable health information.

Several studies have evaluated the reliability of YouTube videos as a source of health information during pregnancy. Studies regarding COVID-19 during pregnancy, gestational diabetes, and epidural analgesia for labor pain identified DISCERN scores of low to moderate information reliability [18,30,31]. Lee et al [32] recently studied the content and quality of the most frequently viewed YouTube videos related to cesarean birth. According to their content-quality analysis, medical videos were of greater quality than nonmedical video sources, and videos describing personal experiences scored significantly lower than other video content. These findings are consistent with our data, which found that while personal vlog videos commonly contained more content, they did not necessarily contain more reliable content.

### Clinical Implications

Uncontrolled postoperative pain may delay hospital discharge and prolong recovery [33]. For birthing individuals, this presents a barrier to independence and caring for a newborn, highlighting the importance of optimizing pain management following cesarean delivery. Experience with pain management interventions, such as enhanced recovery protocols following cesarean, suggests a significant role for thorough education through counseling and written instructions [17]. Additionally, a meta-analysis of emergency room discharge instructions suggested that correct recall may be highest among those who view video discharge instructions [34]. These data suggest a need to translate evidence-based patient education information into a more accessible video format.

While YouTube provides an opportunity to supplement patient education regarding recovery after cesarean birth, current content, including opioid content, is inadequate. Few videos address safe opioid use in the postoperative, outpatient setting, despite campaigns for judicious use. Opioids are known to be prescribed at high rates following cesarean delivery [35]. Fulfillment of postpartum opioid prescriptions and increasing doses are known to increase the risk of serious opioid-related events following cesarean birth [36]. A recent study found a 25% reduction in opioid use when patients viewed an educational video regarding pain management after cesarean delivery [37]. Our content analyses indicate a need to expand upon current YouTube videos to include information regarding opioid use. Improved video content is required for the public to have access to comprehensive information on postcesarean pain management.

YouTube videos provide an opportunity to share quality information on postpartum pain management with a large audience; however, it is essential that clinicians and patients be aware of the limitations of the available videos. This is particularly relevant, as many patients may not discuss the content of electronic sources with their clinicians. A review examining patterns of electronic health use during pregnancy in an underserved, racially diverse population found that while the majority of patients used electronic health sources, approximately 70% of patients discussed their searches with their clinician [38]. Therefore, clinicians may not have an opportunity to discuss the quality of their findings. Interestingly, videos in our study scored low in promoting shared decision-making according to the DISCERN criteria. Even high-quality videos infrequently mention notifying a clinician of warning signs in the postpartum period. Taken together, this highlights the importance of encouraging patients to discuss web-based health information.

### Research Implications

Further research is required to understand how obstetric patients are using YouTube during pregnancy and postpartum. The availability of videos and associated subscribers indicates public interest, but further studies are required to understand the needs of postcesarean individuals as they generate their own YouTube searches. Further work is required to evaluate the information available regarding recovery from vaginal birth as well as pain control in the antepartum, intraoperative, and immediate postoperative periods. This study highlights the need for pain management videos that combine medical expertise with consumer needs. While clinicians should caution patients about the reliability of YouTube videos as a health resource, they should also take an interest in what information their patients are looking for on the internet.

### Conclusions

Patients seeking information from YouTube regarding postcesarean pain management are likely to encounter videos that lack adequate comprehensiveness and reliability. YouTube is an easily accessible resource and an increasingly common source of health information; however, clinicians should counsel patients to use caution when using current YouTube videos as a resource in the postpartum period.

## Appendix

Multimedia Appendix 1 Post-Cesarean Pain Management Search Term.

Multimedia Appendix 2 List of videos (N=73) included in the study.

Multimedia Appendix 3 Discern Score Summary by Video source.

## Abbreviations

REDCap: Research Electronic Data Capture
Vlog: video blog
