# Supplementary material for: Content Quality of YouTube Videos About Pain Management After Cesarean Birth: Content Analysis
Source: JMIR Infodemiology. 2023 Jun 23;3:e40802. doi: 10.2196/40802 (PMC10337243; doi:10.2196/40802)
Supplement: Multimedia Appendix 1 [file infodemiology_v3i1e40802_app1.docx]

**Multimedia Appendix 2**

| **Post-Cesarean Pain Management Search Term** |
| --- |
| 1. Postpartum c section pain 2. Postpartum cesarean pain 3. Postpartum cesarean delivery pain 4. Postdelivery c section pain 5. Postdelivery cesarean pain 6. Postdelivery cesarean delivery pain 7. Postpartum c section pain 8. Postpartum cesarean pain 9. Postpartum cesarean delivery pain 10. Postpartum c section recovery 11. Postpartum cesarean recovery 12. Postpartum cesarean delivery recovery 13. Postdelivery c section recovery 14. Postdelivery cesarean recovery 15. Postdelivery cesarean delivery recovery 16. Postpartum c section recovery 17. Postpartum cesarean recovery 18. Postpartum cesarean delivery recovery 19. Postbirth c section recovery 20. Postbirth cesarean recovery 21. Postbirth cesarean delivery recovery 22. Postbirth c section pain 23. Postbirth cesarean pain 24. Postbirth cesarean delivery pain 25. After birth c section recovery 26. After birth cesarean recovery 27. After birth cesarean delivery recovery 28. After birth c section pain 29. After birth cesarean pain 30. After birth cesarean delivery pain 31. After delivery c section recovery 32. After delivery cesarean recovery 33. After delivery cesarean delivery recovery 34. After delivery c section pain 35. After delivery cesarean pain 36. After delivery cesarean delivery pain |
