# Supplementary material for: Content Quality of YouTube Videos About Pain Management After Cesarean Birth: Content Analysis
Source: JMIR Infodemiology. 2023 Jun 23;3:e40802. doi: 10.2196/40802 (PMC10337243; doi:10.2196/40802)
Supplement: Multimedia Appendix 3 [file infodemiology_v3i1e40802_app3.docx]

**Multimedia Appendix 3**

| Discern score summary by video source. | | |
| --- | --- | --- |
|  | **All Videos**  **(N=73)** | **High-quality**  **(N=22)** |
| **Section 1: Is the publication reliable?** | | |
| 1. Are the aims clear? | 3.0 (0.9) | 3.3 (0.9) |
| 2. Does it achieve its aims? | 3.4 (0.8) | 3.9 (0.5) |
| 3. Is it relevant? | 3.5 (0.8) | 4.1 (0.6) |
| 4. Is it clear what sources were used? | 1.8 (1.0) | 2.0 (1.1) |
| 5. Is it clear when the information used/reported was produced? | 2.1 (0.4) | 2.1 (0.4) |
| 6. Is it balanced and unbiased? | 2.7 (0.9) | 3.0 (0.8) |
| 7. Does it provide detail of additional sources of support and information? | 2.6 (1.1) | 2.8 (1.1) |
| 8. Does it refer to areas of uncertainty? | 2.3 (1.0) | 2.9 (0.8) |
| **Section 2: How good is the quality of the information on treatment choices?** | | |
| 9. Does it describe how each treatment works? | 2.7 (1.1) | 3.3 (0.8) |
| 10. Does it describe the benefits of the treatment? | 3.1 (1.1) | 3.7 (0.8) |
| 11. Does it describe the risks of the treatment? | 1.7 (1.0) | 2.5 (1.1) |
| 12. Does it describe what would happen if no treatment is used? | 1.9 (1.0) | 2.2 (0.9) |
| 13. Does it describe how the treatment choices affect overall quality of life? | 2.6 (1.2) | 3.1 (1.0) |
| 14. Is it clear that there may be more than one possible treatment choice? | 2.5 (1.1) | 3.5 (0.7) |
| 15. Does it provide support for shared decision-making? | 2.6 (0.8) | 3.3 (0.8) |
| **Section 3: Overall rating of the publication** | | |
| 16. Based on the answers above, rate the overall quality? | 2.6 (0.5) | 3.4 (0.5) |
| DISCERN Sum* | 39.2 (8.1) | 46.7 (5.4) |
| Data presented as mean (± standard deviation).  *DISCERN Sum is the sum of DISCERN questions 1-15. | | |
